# A practical discussion to avoid common pitfalls when constructing multiple choice questions items

**DOI:** 10.4103/1319-1683.71992

**Published:** 2010

**Authors:** Eiad A. Al-Faris, Ibrahim A. Alorainy, Ahmad A. Abdel-Hameed, Mohammed O. Al-Rukban

**Affiliations:** *Department of Family and Community Medicine, College of Medicine, King Saud University, Riyadh, Kingdom of Saudi Arabia*; 1 *Department of Radiology, College of Medicine, King Saud University, Riyadh, Kingdom of Saudi Arabia*; 2 *Department of Pathology, College of Medicine, King Saud University, Riyadh, Kingdom of Saudi Arabia*

**Keywords:** Pitfalls, assessment, student

## Abstract

This paper is an attempt to produce a guide for improving the quality of Multiple Choice Questions (MCQs) used in undergraduate and postgraduate assessment. Multiple Choice Questions type is the most frequently used type of assessment worldwide. Well constructed, context rich MCQs have a high reliability per hour of testing. Avoidance of technical items flaws is essential to improve the validity evidence of MCQs. Technical item flaws are essentially of two types (i) related to testwiseness, (ii) related to irrelevant difficulty. A list of such flaws is presented together with discussion of each flaw and examples to facilitate learning of this paper and to make it learner friendly. This paper was designed to be interactive with self-assessment exercises followed by the key answer with explanations.

## INTRODUCTION

Assessment as an important educational tool for both the teacher and the learner is at the top of the agenda for many educators since it is the key to changes in medical education. MCQs are the most frequently used assessment tool worldwide. They have a high reliability per hour of testing and are easy to administer and mark. Without evidence of validity, assessments in medical education have little or no intrinsic meaning. The internal structure of an MCQ item is an important source of validity evidence[1] and therefore necessitates the avoidance of technical item flaws. Such flaws are basically of two main types: (i) related to testwiseness, (ii) related to irrelevant difficulty.[2–5] A list of such flaws is attached [Table 1].

**Table 1:** Checklist for technical MCQ item flaws[3]

Is the question about a trivial concept? If the answer is yes, please cancel the question, No need to proceed and waste your time
Issues related to testwiseness. Is/are – there
Grammatical cue(s). Logical cue(s) i.e. collectively exhaustive options (e.g. hyperkalemia, Normokalemia, Hypokalemia) Absolute terms such as always or never. A correct answer that is longer, more complete and more specific than other options. A word or phrase included in both the stem and the correct answer. Inclusion in the correct answer of most elements in common with the other options.
Issues related to irrelevant difficulty.
The options are long (ideally, the stems should be longer than the options). Numeric data in different formats (i.e. ranges or %) Interaction between two options (e.g. more than 50%; 70%). Vague frequency terms (e.g. rarely, usually, frequently … etc). The options differ in a single continuum or dimension (e.g., which of the following statements concerning disease × is correct). Negatively phrased items (those with except or not) in the lead in statement. None of the above or options (c + d) or (All of the above are correct) is used as an option. Tricky stem or options or numerically complicated. Illogical order of the options (e.g. not in alphabetical sequence).

## OBJECTIVE

With the recent expansion of both the undergraduate and postgraduate medical programs in Saudi Arabia and the wide use of multiple choice questions (MCQs) in assessment, the quality of MCQs have to be improved. This paper is an attempt to do this.

## ISSUES RELATED TO TESTWISENESS

### Grammatical cues

One or more distracters do not follow grammatically from the stem.

#### Example:

The easiest way to get an update on medical information is:

- Books.
- Medical Journals.
- Guidelines.
- The internet.
- Newspapers.

Because an item writer tends to pay more attention to the correct answer than to the distracters, grammatical cues are more likely to occur in the distracters. In this example, testwise students would eliminate a, b, c and e as options because they do not follow grammatically or logically from the stem (plural). Testwise students then have to choose d only, which is the correct answer.

### Logical cues

A subset of the options is collectively exhaustive.

#### Example:

A 20-year-old adolescent attended the A/E complaining of abdominal pain, fatigue, polyurea, polydypsia and vomiting. On examination, the patient was drowsy and dry. Which of following changes you expect to see in the blood test:

- Hypoglycemia.
- Hyperglycemia.
- Normal glucose level.
- Hypocalcemia.
- Metabolic Alkalosis.

In this item, options a, b, and c include all logical possibilities in any patient (a human being has either increased blood sugar, normal blood sugar or decreased blood sugar). The testwise student knows that the correct answer must be A, or b, or c whereas the non-testwise student spends time considering d and e.

### Use of absolute terms

Terms such as “always” or “never” are used in options.

#### Example:

In deep venous thrombosis (DVT) of the lower extremity, which of the following statements is the MOST appropriate?

- 70% of patients will have physical findings on examination.
- The gold standard test for the diagnosis of DVT is the radiofibronogen leg scanning.
- Anticoagulant is the treatment of choice for distal lower extremity DVT.
- Pulmonary emboli always come from lower extremity DVT.

Testwise students usually do not select items with the words (always or never like item d) and instead they tend to choose items with the words (usually or frequently) knowing that nothing is absolute in medicine.

### Long correct answer

Correct answer is longer, more specific, or more complete than other options.

#### Example:

The parent of a 2-year-old boy reported that he shows in toeing when walking. On examination, the child exhibits femoral anteversion. The MOST appropriate treatment is:

- Reassuring to the parents that the condition usually corrects itself as the child grows older.
- Referral to an orthopedist.
- Referral to a physical therapist.
- Bracing to correct internal rotation of the femurs.

In this item, option A is longer than the other options and it is the correct one. Item writers tend to pay more attention to the correct answer than to the distractors. Because you are teachers, you write long correct answers that include additional instructional material, parenthetical information, caveats, etc. Sometimes this can be quite extreme.

### Word repeats

A word or phrase is included in the stem and in the correct Answer.

#### Example[3]

A 58-year-old man with a history of heavy alcohol use and previous psychiatric hospitalization is confused and agitated. He speaks of experiencing the world as unreal. This symptom is called:

- Depersonalization.
- Derailment.
- Derealization.
- Focal memory deficit.
- Signal anxiety.

This item uses the word “unreal” in the stem, and “derealization” is the correct answer. Sometimes, a word is repeated only in a metaphorical sense, e.g., a stem mentioning bone pain, with the correct answer beginning with the prefix “osteo”.

### Convergence strategy

The correct answer includes the most elements in common with the other options.

#### Example:

Oral Polio Vaccine (OPV) induces specific:

- Active, artificial, congenital immunity.
- Active, artificial, acquired immunity.
- Passive, artificial, acquired immunity.
- Active, natural, acquired immunity.
- Passive, natural, acquired immunity.

The correct answer is the option b. The underlying premise is that the correct answer is the option that has the most elements in common with the other options; it is not likely to be an outsider.

## ISSUES RELATED TO IRRELEVANT DIFFICULTY

Several flaws introduce irrelevant difficulty into the MCQ item not necessary in assessment.

### Options are long, complicated, or double

#### Example:

A team of health care planners wishes to estimate the prevalence and incidence of AIDS in a particular community, which of the following is the most appropriate:

- The prospective cohort study design is the most suitable for estimating the prevalence of AIDS.
- The cross-sectional study design is the most suitable for estimating incidence of AIDS and the study group should represent not less than 30% of the community under study.
- Incidence is estimated as the proportion of study subjects, who test positive for AIDS from a random sample of individuals selected from the community.
- Information on the incidence of AIDS helps to plan and evaluate health care needs and services to assess the health care burden imposed on the community.
- A difference between the incidence rates in two groups of subjects exposed and not exposed to a risk factor may be used to test the relative strength of the association between the factor and the occurrence of AIDS.

The options are very long and complicated. Trying to decide among these options requires a significant amount of reading because of the number of elements in each option. This can shift what is measured in an item from knowledge of the content to reading speed. The second flaw in this item is that only options a and b follow logically from the stem, while options c, d, and e are not directly related to the purpose of the health care planners in the stem. Careful look to option c shows that there are two facts (double facts) that need to be judged by the student. Double facts in an option introduce difficulty when one fact is true and the other is wrong. As a rule, if you need to test knowledge of two facts each one should be made into an option.

### Numeric data are not stated consistently

When numeric options are used, they should be listed in numeric order and in a single format (i.e., as single terms or as ranges). Confusion occurs when formats are mixed and when the options are listed in an illogical order or in an inconsistent format.

#### Example

A 24-year-old woman delivered a boy with Down syndrome; she is depressed and asks you: what is the likelihood of her having another child with a similar condition? Your answer will be:

- 2%
- 5%
- 25%
- Less than 10%
- 15 to 18%

In this example, Options d and e are expressed as ranges (not specific percentages). All options should be expressed either as ranges or as specific percentages. Absolute numbers should be used when there is consensus on their value. Otherwise, it is better to use ranges to avoid differences between sources that may confuse the student. In addition, the range for option d includes options a and b, which is not advisable.

### Frequency terms in the options are vague (e.g. rarely, usually)

Research has shown that vague frequency adverbs are not consistently defined, even by experts.

#### Example

A one month old infant presents with sudden onset of paroxysms of loud crying lasting several hours, a tense abdomen, drawn up legs, cold feet and clenched fists. Which of the following is a true statement about his problem:

- It is caused by excessive swallowing of air.
- It is due to cow’s milk protein allergy.
- It is related to insufficient fluid intake.
- It rarely lasts beyond 3 months of age.
- Sedation is usually effective therapy.

In this example, the definitions of the words “rarely” and “usually” used in options d and e, respectively should be stated or replaced with numbers.

### Options are not parallel (heterogeneous)

The options following a stem belong to different categories. This is one of the frequently encountered mistakes.

#### Example

A 75-year-old woman complains of severe neck and shoulder pain. You suspect polymyalgia. Which one of the following is the MOST appropriate?

- Shoulder girdle muscle tenderness is not related to the condition.
- Loss of weight and fever would be expected.
- Her urine is likely to contain Bence-Jones protein.
- Muscle biopsy is positive if the diagnosis is correct.
- Steroids produce a very prompt symptomatic response but must be continued for up to 3 months in this condition.

The response options in this example are not parallel. The options a and b are about clinical features and options c and d are about laboratory investigations, while option e is about management. The options should be homogenous.

### Options are not in a logical order

It is a good practice to be consistent in the use of alphabetical order. Whenever options are in spectrum, it is preferable to list them in either a logical order or alphabetically. For instance, numeric options should be listed in ascending or descending order. Logical order is more convenient for the students and eliminates human bias.

#### Example

End arteries are seen in the:

- Adrenals
- Testicles
- Thyroid gland
- Thymus
- Brain

In this case, it is easier to follow and read if the options are arranged from top down in this order: brain, thyroid, thymus, adrenals, and then testicles.

### Negatively phrased stem

It is not advisable to use a negative statement in the stem, particularly if one or more of the options contain negatives (double negative). It is confusing for the examinee and serves us no purpose.

#### Example

Which of the following is NOT a feature of neurofibromatosis?

- Skin pigmentation
- No increased risk of brain tumors
- Hearing loss
- No renal anomalies
- Positive family history

While the stem is negative, options b and d are also negative.

### “None of the above” or “all of the above” or “option a + b” are used as an option:

The phrase “None of the above” is problematic in items where judgment is involved and where the options are not absolutely true or false. Use of “none of the above” as the true option may turn the item into a true/false item; each option has to be evaluated as more or less true than the universe of unlisted options.

#### Example

Chicken pox is caused by the same virus that causes:

- Herpes simplex labialis
- Herpes zoster
- Herpetic stomatitis
- All of the above
- None of the above

If the student knows that options a and b are true but has no idea about option c, it is easy to figure out that the correct answer is d.

### Stems are tricky or unnecessarily complicated

Sometimes, item writers can take a perfectly easy question and turn it into something so convoluted that only the most determined student will read it. The following item is a sample of that kind of item. An item are sometimes complicated by the use of uncommon abbreviations that are not spelled out.

#### Example

You suspect acute infectious mononucleosis due to Epstein-Barr virus in a 3-year-old child. The laboratory results MOST consistent with this diagnosis would be:

VCA = Viral Capsid Antigen, IgM, IgG = Immunoglobulin M or G, EBNA = Epstein-Barr Nuclear Antigen.

Anti-VCA=1, Anti-VCA (IgM) =2. Anti-EBNA (IgG) =3

- 1=Negative, 2=Negative, 3=Positive
- 1=Negative, 2=Negative, 3=Negative
- 1=Negative, 2=Positive, 3=Positive
- 1=Positive, 2=Negative, 3=Positive
- 1=Positive, 2=Positive, 3= Negative

## SELF ASSESSMENT EXERCISE OF MCQ ITEM FLAWS

Self-assessment is a valuable educational tool. It has been given a higher profile in the last few years because of the switch of emphasis towards more self-directed learning and the popularity of distance learning. The following is a self-assessment exercise to help recognize poorly constructed MCQs. Read the questions and list the problems before looking at the correct answer. Table 1 may be found helpful as a checklist and reminder. It must be noted that this exercise does not intend to cover all possible flaws. The discussion section will concentrate on the major critiques. There may be problems in some questions that are not addressed in the discussion.

1. DOTS is the most effective treatment in:

- HIV/AIDS.
- Leishmaniasis.
- Malaria.
- Schistosomiasis.
- Tuberculosis.

2. A 2-year-old girl brought to the A/E because of several thin-walled, 1 to 2-cm “blisters” on her face and several crusted, honey-colored and weeping lesions on her arms. The patient is febrile and has no associated lymphadenopathy. Which of the following statements concerning the most likely etiology for these findings is CORRECT?

- Acute glomerulonephritis occurs in 20% of patients.
- Acute rheumatic fever is a common complication.
- Antibiotic therapy should cover staphylococci and streptococci.
- Penicillin is the treatment of choice.
- Oral corticosteroids are first line therapy.

3. The patient subsequently has an exercise tolerance test which reveals a 2.5mm ST segment depression at METS of activity as the patient achieved 50% of his age-predicted maximum heart rate. Which of the following statements regarding this test result is (are) TRUE?

- It probably indicates angina pectoris.
- Coronary angiography is indicated.
- A hypotensive response with stress testing suggests severe ischemia and severe (probably multi-vessel) coronary artery disease.
- a and b.
- All of the above statements are true.

4. Which of the following is (are) a clinical hallmark of asthma?

- Cough.
- Nocturnal dyspnea.
- Shortness of breath.
- Wheezing.
- a, c and d.

5. In Epidemiology

- Analytical epidemiology describes frequency of disease occurrence in relation to person, place, and time.
- The incidence rate is the number of old and new cases in the population during a specific time interval.
- The epidemic concept involves infectious diseases only.
- Adjusted rates are commonly used to correct for age differences.
- The most satisfactory procedure to determine association between variables is by observational studies.

6. Regarding chronic otitis media the following are true except:

- Cases with peripheral (postero-superior) perforation are at a higher risk of complication than cases with central perforation.
- May result in permanent deafness.
- Discharge could be of bad odor.
- Tympanoplasty is needed in all cases.
- Cholesteatoma is a dangerous complication.

7. Which of the following statements about Polymyalgia rheumatica (PMR) is true?

- PMR can affect any age-group, but is most often seen in geriatric patients.
- PMR responds to corticosteroids treatment within several days.
- If untreated, the symptoms of PMR will spontaneously resolve within 2 weeks.
- Muscle weakness is found on physical examination.
- Abdominal symptoms are present.

8. A 17-year-old lady is brought into your office with a 3-day history of nausea, vomiting, generalized abdominal pain and lethargy. She appears acutely sick. On examination, the patient’s blood pressure is 170/70 mmHg, and her pulse is 140 and regular. Her respirations are 45/minute and regular. She has no history of significant disease. Her random blood sugar is 40 mmol/L (720 mg%). Her urine has +4 ketones. Which of the following statements is (are) TRUE of this patient?

- This patient is at risk for going into hyperosmolar diabetic coma.
- This patient should be treated with intravenous fluids, intravenous insulin, and intravenous potassium.
- This patient can be safely treated as an outpatient.
- a and b.
- b and c.

9. A 51-year-old lady who is worried about developing osteoporosis because her elderly mother broke her hip a month ago following minor injury. She is still having irregular and scanty periods and she is not too keen on the idea of taking hormones. The most appropriate course of action is:

- Order bone densimetry.
- Prescribe HRT.
- Give multi-vitamins and calcium.
- Counsel the patient about the advantages and disadvantages of HRT for the prevention of osteoporosis.

10. Primary dysmenorrhea:

- Begins 7-10 days before menses.
- Is associated with ovulatory cycles.
- Is caused by organic disease.
- Occurs around six months after menarche.
- Can be treated with a steroid.

11. Hepatitis A infection:

- Homosexuals are risky groups.
- Difficult to be prevented by immunization.
- Is a foeco-orally transmitted disease.
- Associated cirrhosis in 1-3%
- Incubation period=2-5 days

12. The highest fatality rate in tetanus ate is seen in which of the following age groups:

- Young adults.
- Neonates.
- Infants.
- Females
- Old people.

13. A lucid interval is a period of:

- Amnesia.
- Concussion.
- Consciousness.
- Unconsciousness

14. Which of the following changes is NOT usually seen in a 30-year-old obese patient treated with orlistat (xenical)?

- Decrease in total serum cholesterol level.
- Decrease in LDL cholesterol level.
- Decrease in fat soluble vitamin level.
- Changes in coagulation parameters in patients also taking warfarin by increasing vitamin K absorption.
- Steatorrhea, flatus, fecal incontinence and oily spotting.

15. The incidence of Bronchial Asthma among schoolchildren in Kingdom of Saudi Arabia (KSA) was found to be:

- About 20 percent.
- About 7 percent.
- About 20-30 percent.
- About 7-10 percent.
- More than 18 percent.

## ANSWERS TO THE EXERCISE

To avoid repetition and to emphasis this important point, it should be noted that questions numbered 1, 4, 5, 6, 7, 10, 11, 12, 13 and 15 are traditional (context free) questions. This type of question is not advisable.

### Question 1

- This is a single correct MCQ rather than single best answer because options are either 100% true or 100% false.
- The item tests recall of knowledge rather than application of knowledge. This problem could be avoided by using clinical vignettes or patient scenarios.
- Note that the abbreviation “DOTS” has not been spelled out. It stands for “directly observed therapy short course”. This is a common mistake. It represents irrelevant difficulty unless the aim of the question is to test the student’s knowledge in standard abbreviations.

### Question 2

- Options are not consistent with the lead in statement. None of the options mentioned the most likely etiology in this case.
- The options are not homogenous, one of them is about etiology, the second is about complication and the others “management”.
- This is a true/false rather than single best answer question.

### Question 3

- The paragraph “the patient subsequently…” indicates that this question is a continuation of a previous question that was not shown here.
- “All of the above statements are true” and “a and b” options are used. This is one of the flaws discussed earlier.
- Option “c” is much longer and more detailed than other options.
- The options are not homogenous.

### Question 4

- The item tests recall of knowledge (stimulus free) as there is no patient scenario for the application of knowledge.
- Option “e” is a combination of multiple options. If the student knows two options as correct, the inevitable choice would be “e”.
- It is clear that “(are)” has been introduced to avoid grammar non-matching of option “e”.
- The stem that contains singular and plural forms is difficult to read.

### Question 5

- The shape of the items is incorrect. The options are longer than the stem.
- There is no lead-in question (statement).
- Use of vague words e.g. commonly in d.
- Options are not homogenous.

### Question 6

- Negatively phrased item used in the stem (true except). It is better to use positively stated stem.
- Options are not homogenous (complications, signs, managements).
- Use of absolute terms (all in option “d”), the testwise student knows it is most likely false.
- Option “a” is much longer than others.

### Question 7

- The item tests recall of knowledge (stimulus free) rather than application of knowledge.
- Options are not homogenous (signs, symptoms, management and prognosis).
- Uses vague descriptions (most often).

### Question 8

- The correct answer (b) is longer, more complete and more specific than other options.
- “a and b” and “b and c” are used in the options. This is one of the flaws.

### Question 9

- It is a stimulus rich question. It consists of homogenous options which is good.
- Option “b” is not logical because the stem indicates that the patient is not keen on this option.
- The correct answer (d) is longer, more complete and more specific than other options.
- Abbreviations should be avoided e.g. “HRT”.
- The correct answer may be controversial is it bone densimetry/HRT or Fosamax, Ca and phosphate? If there is any controversy among experts, the question should not be included in the exam.

### Question 10

- The stem does not contain question or lead-in statement. This is the classical problem when the item writer tries to convert true/false question to single best answer by making only one option true or false. The result will be a single correct answer question (not single best) that tests factual recall.
- Options are not homogenous (etiology/clinical features/treatment).

### Question 11

- Grammatical cue, testwise students would choose option “c” because it is the only one that completes the sentence.
- Options are not homogenous.
- No lead-in statement.

### Question 12

- This is a single correct MCQ rather than single best.
- Options are not in logical order. It is better and more convenient for the student to list options (age groups in this case) in ascending or descending order.
- Not homogenous (sex and age)
- The age groups are exhaustive; therefore, option d can be excluded.

### Question 13

- This is a single correct MCQ rather than single best.
- Logical cue (i.e. collectively exhaustive options) the correct answer either “c” or “d” because they included all possibilities.

### Question 14

- Option “d” is longer, more complete and more specific than other options.
- Negatively stated question stem.
- Option “e” does not follow grammatically from the stem. Stem is in singular form and option “e” contains multiple possibilities.

### Question 15

- Numeric data are not in order or in a consistent format. Options c, d and e are expressed as ranges, whereas Options a and b are specific percentages.
- The range for option e includes options a and c, and the range for option d includes option b
